# Degeneration of Retinal ON Bipolar Cells Induced by Serum Including Autoantibody against TRPM1 in Mouse Model of Paraneoplastic Retinopathy

**DOI:** 10.1371/journal.pone.0081507

**Published:** 2013-11-25

**Authors:** Shinji Ueno, Koji M. Nishiguchi, Hidetoshi Tanioka, Atsushi Enomoto, Takashi Yamanouchi, Mineo Kondo, Testuhiro R. Yasuma, Shunsuke Yasuda, Noriyuki Kuno, Masahide Takahashi, Hiroko Terasaki

**Affiliations:** 1 Department of Ophthalmology, Nagoya University Graduate School of Medicine, Nagoya, Japan; 2 Research and Development Center, Santen Pharmaceutical Co., Ltd., Ikoma, Japan; 3 Department of Pathology, Nagoya University Graduate School of Medicine, Nagoya, Japan; The University of Melbourne, Australia

## Abstract

The paraneoplastic retinopathies (PRs) are a group of eye diseases characterized by a sudden and progressive dysfunction of the retina caused by an antibody against a protein in a neoplasm. Evidence has been obtained that the transient receptor potential melastatin 1 (TRPM1) protein was one of the antigens for the autoantibody against the ON bipolar cells in PR patients. However, it has not been determined how the autoantibody causes the dysfunction of the ON bipolar cells. We hypothesized that the antibody against TRPM1 in the serum of patients with PR causes a degeneration of retinal ON bipolar cells. To test this hypothesis, we injected the serum from the PR patient, previously shown to contain anti-TRPM1 antibodies by westerblot, intravitreally into mice and examined the effects on the retina. We found that the electroretinograms (ERGs) of the mice were altered acutely after the injection, and the shape of the ERGs resembled that of the patient with PR. Immunohistochemical analysis of the eyes injected with the serum showed immunoreactivity against bipolar cells only in wild-type animals and not in TRPM1 knockout mice,consistent with the serum containing anti-TRPM1 antibodies. Histology also showed that some of the bipolar cells were apoptotic by 5 hours after the injection in wild type mice, but no bipolar cell death was found in TRPM1 knockout mice, . At 3 months, the inner nuclear layer was thinner and the amplitudes of the ERGs were still reduced. These results indicate that the serum of a patient with PR contained an antibody against TRPM1 caused an acute death of retinal ON bipolar cells of mice.

## Introduction

Light stimulation of the rod and cone photoreceptors elicits signals that are transmitted to the bipolar cells and then to the retinal ganglion cells (RGCs). At present, there are many retinal diseases that are caused by a degeneration of the photoreceptors or the RGCs. Retinitis pigmentosa is an example of the former type of diseases and is caused by a degeneration of the rods followed by the cones. Glaucoma is an example of the second type of diseases that is caused by the death of RGCs. There is no known retinal disease caused by bipolar cell degeneration.

The paraneoplastic retinopathies (PRs) are a group of diseases characterized by a sudden and progressive decrease in the function of the retina. The retinopathies have been shown to be caused by a circulating anti-retinal autoimmune antibody against a protein of a neoplasm [1-4]. One subtype of the PRs has been reported to be caused by an autoantibody against a protein expressed by retinal ON bipolar cells [5,6]. The symptoms and signs of these patients were a sudden onset night blindness, photophobia, and a decrease of the visual acuity. The electroretinograms (ERGs) elicited by a standard flash stimuli had a selective reduction of the b-waves with normal a-waves. This resulted in a waveform called a negative type ERG which suggested a dysfunction of the ON bipolar cells. Additional ocular examinations including fundus examination showed no distinctive features [6]. Originally these diseases were reported in patients with melanomas, and they were named melanoma-associated retinopathies (MARs) [7,8]. However, it has been reported that neoplasms other than melanomas can cause the bipolar cell dysfunction [5,9].

We and others have recently shown that the transient receptor potential melastatin 1 (TRPM1) was an antigen for the autoantibody against the ON bipolar cells in some patients with PR [10,11]. TRPM1 is a protein associated with the ion-conducting plasma membrane channels that mediates the light responses of ON bipolar cells [12-14]. Several studies have reported the presence of neural degeneration in the paraneoplastic syndrome including other types of paraneoplatic retinopathies [4,15-17], but none have shown that the serum of patients with PR can cause a degeneration of the retinal ON bipolar cells.

Thus, the purpose of this study was to determine whether the serum of a PR patient with the TRPM1 antibody will cause a degeneration of ON bipolar cells. To achieve this, we injected serum from a PR patient who had an autoantibody against TRPM1 [11] into the vitreous of mice and evaluated its effects on retinal function and histology. We show serum including autoantibody against TRPM1 caused acute retinal ON bipolar cell degeneration.

## Materials and Methods

### Animals

All experimental procedures adhered to the ARVO Statement for the Use of Animals in Ophthalmic and Vision Research and the guidelines for the Use of Animals at the Nagoya University School of Medicine. Nagoya University Animal Experiment Committee approved this project (approval number 24456). Seventy C57BL/6 mice at 7-10 weeks-old-age were used. TRPM1 knock-out mice were kindly given to us by Dr. T. Furukawa of Osaka Bioscience Institute [14].

### Human

The Nagoya University Hospital Ethics Review Board approved this study (approval ID 1131). The procedures used conformed to the tenets of the Declaration of Helsinki of the World Medical Association. A written informed consent was obtained from the patient after he was provided with sufficient information on the procedures to be used. 

### Sera and intravitreal injections

Sera were collected from one PR patient and one visually normal male subject. The patient had lung cancer and the negative type ERG, and his eye phenotype has been described in detail [11]. Mice were anesthetized with ether, and 1 µL of the serum of the patient or control subject was injected intravitreally into C57BL/6 mice. Other C57BL/6 mice had l-2 amino-4-phosphonobutyric acid (APB; Sigma-Aldrich, St. Louis, MO) solution injected into the vitreous. The APB was dissolved in sterile saline, and the intravitreal concentration was estimated to be 1 mM. The injections were made with a glass micropipette with a microinjection apparatus (IM 300 microinjection; Narishige, Tokyo, Japan). 

### Electroretinograms (ERGs) of mice

To evaluate the function of the retina after the intravitreal injection of the sera and APB, five C57BL6 mice were injected with the PR patient’s whole serum in one eye and the serum of the control subject in the other eye. ERGs were recorded at 3 hrs, 3 days, 1 month, 3 months, and 6 months after the injection. We also recorded ERGs from 6 mouse eyes 3 hours after the intravitreal injection of APB solution to determine the effect of blocking the ON bipolar cell on the ERGs [18]. The procedures used for the ERG recordings have been described in detail [19]. Scotopic ERGs were elicited by stimulus intensities of -2.6 and 1.0 log cd-s/m^2^ after one hour of dark-adaptation, and the photopic ERGs were elicited by a stimulus intensity of 1.0 log cd-s/m^2^ presented on a rod saturating background of 40 cd/m^2^. 

### Western blot analyses

The cDNAs for human and mouse TRPM1 were generously provided by Dr. T. Furukawa of the Osaka Bioscience Institute, and Western blots were performed as described [11]. HEK293FT cells (Invitrogen, Carlsbad, CA) were grown and transfected with control plasmids (Flag-GST), mouse TRPM1 (mTRPM1), or human TRPM1 fused to the 3xFlag epitope at the carboxyl terminus (hTRPM1-Flag). The antibodies used included anti-mouse TRPM1 (1:100), anti-Flag (1:1000; Sigma, St Louis, MO), and anti-β-actin (1:5000; Sigma).

### Immunohistochemical analyses

Eyecups from mice were fixed in 4% paraformaldehyde in phosphate-buffered saline (PBS) for 1 hour and placed in 30% sucrose in PBS overnight at 4° C. The eyecups were embedded in OCT compound (Tissue-Tek; Sakura Finetek Japan Co. Ltd., Tokyo, Japan), and 12-18 μm thick frozen sections were cut along the radial axis of the eye. After the sections were permeabilized in 0.1% Triton X-100 in PBS for 15 minutes, they were blocked in 4% goat serum in PBS for 30 minutes. They were then incubated with primary antibodies for 1 hour and for another hour in a mixture of secondary antibodies and diamino-2-phenyl-indol (DAPI; Molecular Probes, Life Technologies, Carlsbad, CA). The primary antibody was omitted for the immunostaining of anti-human IgG. Rabbit anti-protein kinase C α subunit (PKCα) (1:500 Sigma-Aldrich; St Louis,MO) and rat anti-F4/80 (1:400; AbD Serotec, Oxford,UK) were used as the primary antibodies. Goat anti-human-IgG-Alexa488, goat anti-rabbit-IgG-Alexa488, and goat anti-rat-IgG-Alexa488 (Molecular Probes; Life Technologies; Carlsbad, CA) were used as the secondary antibodies and were diluted by 1:500 in PBS. 

### TdT-mediated dUTP-biotin nick-end labeling (TUNEL) staining

To detect apoptotic cells, the sections were stained with an *in situ* apoptotic cell detection kit (Click it TUNEL Alexa Fluor 488, Invitrogen). TUNEL staining was performed on retinal sections obtained 1 day after the serum injection. The number of TUNEL-positive cells in the INL was counted in three independent images (180 x 240 μm) from one eye and averaged. Three eyes that were injected with the control serum and three eyes injected with the patient’s serum were examined. The number of DAPI-positive nuclei in the INL was also counted.

### Transmission Electron Microscopic (TEM) Examinations

Three eyes from 3 mice injected intravitreally with the serum from the control subject and three eyes with the serum from the patient with PR were enucleated after 5 hours, 3 days, and 3 months. The eyes of two TRPM1 knockout mice of 9-weeks-of-age were obtained at 5 hours after the injection of the patient’s serum. All of the eyes were fixed in 2.5% glutaraldehyde in 0.1 M PBS, washed 3 times in PBS, and postfixed for 1 hour in 1% aqueous osmium tetroxide. They were then dehydrated in a graded ethanol series, transferred to QY-1 (Nissin EM, Tokyo, Japan), and embedded in Quetol-812 (Nissin EM, Tokyo, Japan). Semi-thin sections were cut and stained with 0.05% toluidine blue for light microscopy (LM). Ultrathin sections were stained with uranyl acetate and lead citrate and examined with a TEM (H-7600; Hitachi, Tokyo, Japan). 

### Hematoxylin-eosin (HE) staining

Three months after the intravitreal injection of the serum from the control subject into 6 eyes and serum from the PR patient into 6 eyes of C57BL6 mice, the eyes were enucleated and fixed overnight in a mixture of 10% neutral buffered formalin and 2.5% glutaraldehyde. The eyes were transferred to 10% formalin. The tissues were embedded in paraffin, sectioned vertically through the optic nerve so that the superior and inferior halves could be examined. The mounted sections were stained with hematoxylin and eosin (HE). The thickness of the combined inner nuclear layer (INL) and outer plexiform layer (OPL) and outer nuclear layer (ONL) were measured every 400 μm across both the superior (S1-3) and inferior hemispheres (I1-3). We calculated the ratio of the thickness of the INL+OPL/ONL to reduce any artifacts produced during the processing of the tissues.

### Photography

HE and toluidine blue stained sections were photographed with a Nikon Eclipse TE 2000 microscope or with a Digital sight DS-U1. For the immunohistochemical analyses and TUNEL assays, photographs were taken with a confocal D-Eclipse C1 microscope (Nikon, Tokyo,Japan). Photomicrographs were from the retina near the post pole, and images of immunohistochemical analysis of PKCα were taken from the peripheral to mid peripheral retina.

### Statistical analyses

Student's *t* tests were used to determine the significance of any differences in the thickness of the retinal layers and evaluation of the ERG amplitudes. A P <0.05 was considered significant. 

## Results

### Reduction of scotopic b-wave and relative preservation of a-wave in ERG induced by itravitreal injection of patient’s serum in mice

**Figure 1:**
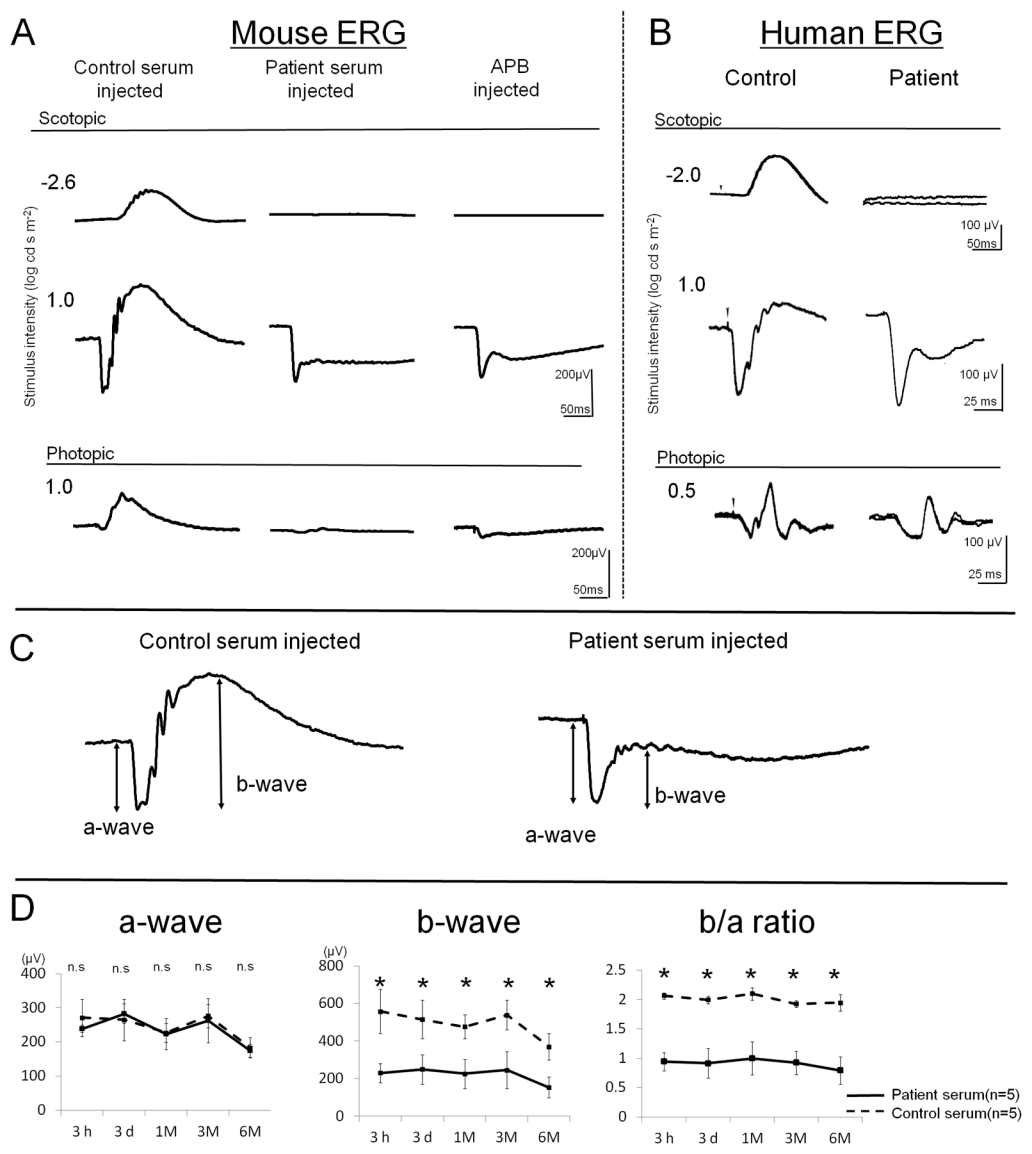
Electroretinograms (ERGs) recorded after an intravitreal injection of serum. (A) Representative Ergs of mice that received an intravitreal injection of serum from a normal control subject, the serum of a PR patient and APB solution. (B) For comparison, ERGs of a control subject and the patient are shown. ERGs were elicited under 3 conditions; scotopic dim flash, scotopic bright flash, and photopic bright flash. (C) Representative Ergs recorded 3 days after the injection of the control and patient sera into the vitreous. The ERGs were elicited by 1.0 log cds m^-2^ for the scotopic condition. The a-wave was measured from baseline to first negative trough, and the b-wave was measured from the bottom of a-wave to the peak of the following positive wave as indicated by the arrows. (D) Amplitudes of a-wave, b-wave, and b/a ratio recorded at 3 hours, 3 days, 1 month, 3 months, and 6 months after the serum injection are plotted (mean ± SEM ,n=5; *P<0.05 ).

Representative Ergs recorded from eyes 3 days after the intravitreal injection of the serum from a control subject and the serum from the PR patient are shown in Figure 1A. Also shown are the ERGs recorded 3 hours after the intravitreal injection of APB. The ERGs of the eye that received the control serum has a positive b-wave of about 200 μV that was elicited by a dim flash under scotopic conditions(-2.6 log cd-s/m^2^). With a bright flash of 1.0 log cd-s/m^2^, the ERG consisted of a negative a-wave of about 300 μV and a b-wave of about 600 μV under scotopic conditions. Under photopic conditions, the ERG consisted of a small a-wave and a b-wave of about 200 μV. In contrast, the ERGs of the eye that received the patient’s serum elicited by a scotopic dim flash was almost extinguished. With a bright flash, the a-wave amplitude was similar to that of the control but the b-wave was markedly reduced. This resulted in a negative type ERG (a-wave>b-wave). The b-wave amplitudes of the photopic ERGs were also reduced. The ERGs after APB injection had almost the same pattern of ERGs recorded after the patient serum injection.

The ERGs recorded from the PR patient and from a control subject are shown in Figure 1B. These ERGs were recorded under approximately the same conditions as the ERGs in the mice. The ERGs of the patient resembled very closely the ERGs of the mouse that had received the patient’s serum; almost non-recordable b-wave with a dim flash and a negative waveform ERG with a bright flash under scotopic conditions. Under photopic conditions, the ERG of the patient had large wide a-wave and small and delayed b-wave. Although the waveform resembled that of non-human primates intravitreally injected with APB to block the function of ON bipolar cells, the result seemed to oppose to that of the mouse ERGs that were markedly reduced (see discussion) [20,21]. 

The a-wave was measured from the baseline to the first negative trough and the b-wave was measured from the bottom of the a-wave to the peak of the following positive wave (Figure 1C).　We followed the ERGs of 5 mice for up to 6 months after the injection of the patient’s serum in one eye and the control serum in the other eye. We measured the amplitudes of the a- and b-waves elicited by the scotopic bright flashes (1.0 log cd-s/m^2^). Because the a-waves originate from photoreceptor activity [22-24], they were used to assess the function of the photoreceptors. In the same way, the b-waves originate from ON bipolar cells, and they were assessed to estimate the function of the ON bipolar cells [25]. We also used the b-/a-wave amplitude ratio of the scotopic bright flash ERGs to assess the functioning of the postsynaptic neurons. A low b-/a-wave ratio would indicate reduced postsynaptic activity relative to that of the photoreceptors. 

The mean amplitude of a-wave of the two types of eyes was not significantly different at all time points. However, the amplitude of the b-wave and b-/a-wave ratios were reduced after 3 hours and did not recover for at least 6 months after the injection (Figure 1D). These results indicated that the function of the ON bipolar cells was depressed within 3 hours after the injection of the patient’s serum, and the reduction persisted for at least 6 months. 

### Autoantibody against TRPM1 in patient’s serum

**Figure 2:**
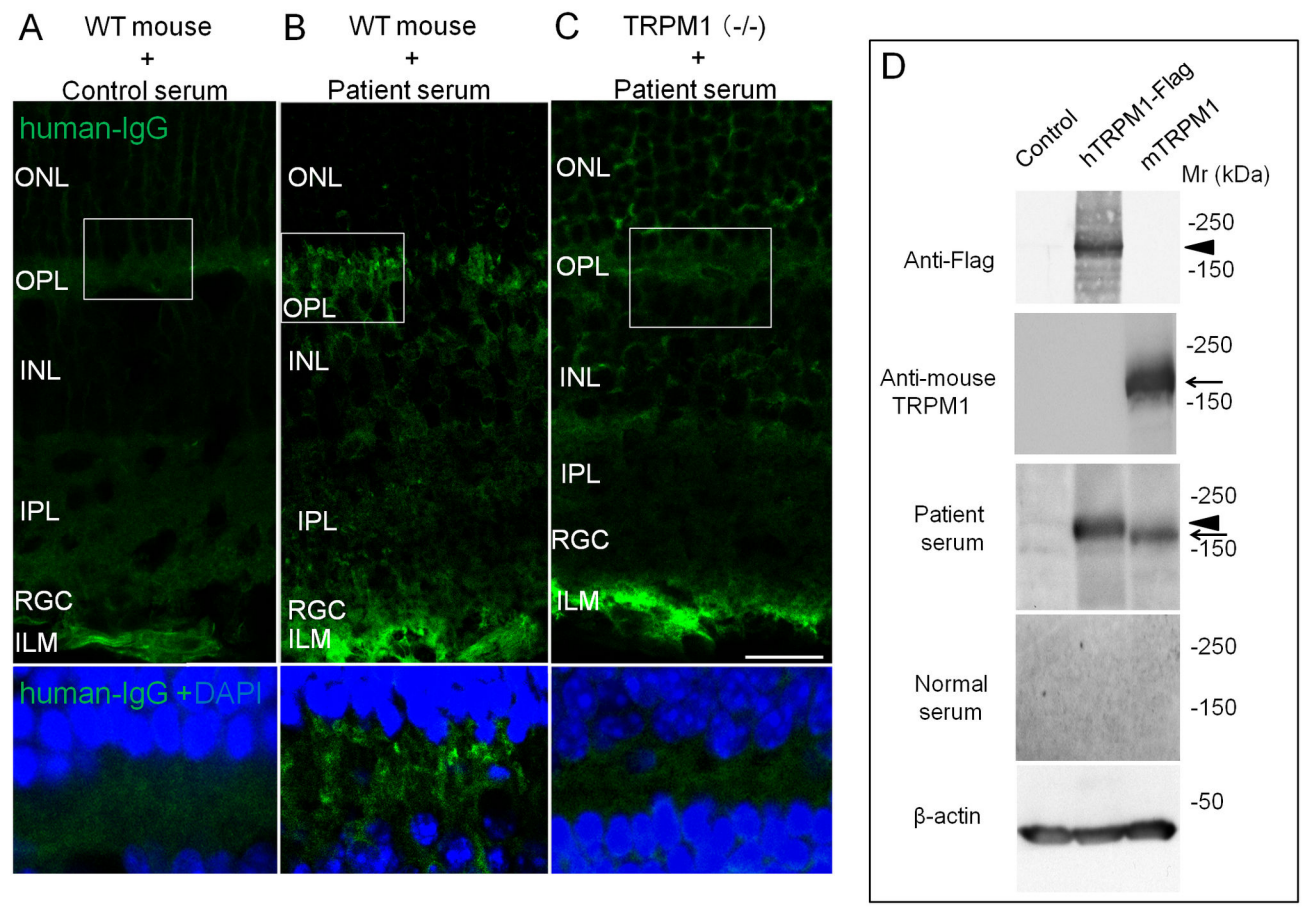
Immunostaining with anti-human IgG antibody of mouse retina obtain 5 hours after intravitreal injection of serum. Immunostaining of mouse retina that was injected with control serum (A), with the patient's serum (B), and immunostaining of TRPM1 knockout mouse retina with the patient's serum (C). Human IgG was stained green. The regions indicated by the white boxes are enlarged below and stained with DAPI in blue (bottom of A, B and C). Fluorescein staining is prominent in the OPL (B). The scale bar is 25 μm in panels (A) to (C). (D) Immunoblots of transfected cell lysates using an antibody against Flag tag, antibody against mouse TRPM1, serum from PR patient, and serum from control subject. Arrowheads indicate the TRPM1-Flag protein bands and arrow indicates mouse TRPM1 protein. β-actin (β-act) was used for loading control. Patient serum had autoantibodies against both mouse and human TRPM1. Abbreviations: ILM - inner limiting membrane, RGC - retinal ganglion cells, IPL-inner plexiform layer, INL - inner nuclear layer, OPL - outer plexiform layer, ONL - outer nuclear layer.

To confirm that the injected IgG in the patient serum was against TRPM1, we immunostained the retinas obtained 5 hours after the intravitreal injection of the serum with anti-human IgG, a secondary antibody. The immunohistochemical findings of the retina from a mouse that received the control serum, the retina of a mouse that received the patient’s serum, and the retina of a TRPM1 knockout mouse that received the patient’s serum are shown in Figures 2A-2C. In the TRPM1 knockout mouse, no reduction of the retinal ON bipolar cell markers including Chx10, Goα, and mGluR6 except TRPM1 was observed and the retinal morphology appeared to be normal [14]. Because some of the IgG in the injected serum was retained in the vitreous and inner limiting membrane (ILM), the ILM were stained in the three types of mice (Figures 2A-2C). Punctate staining was present in the OPL in the mouse after the injection of the patient’s serum (Figures 2B), but only weak uniform background staining was detected in the other two retinas (Figures 2A and 2C). The distribution of these punctate staining agreed with the report of immunostaining of TRPM1 on the dendritic tips of the retinal ON bipolar cells [14]. The absence of staining in the TRPM1 knock out mouse strongly suggested that the antibody was against TRPM1 or a protein whose expression was dependent on TRPM1. 

We also performed Western blots to confirm that the patient’s serum would recognize both human and mouse TRPM1. We transfected HEK293FT cells with expression plasmids containing control, human TRPM1-Flag (hTRPM1-Flag), or mouse TRPM1 (mTRPM1). Western blot analysis was performed on whole cell extracts, and we confirmed that a hTRPM1-Flag band and a mTRPM1 band were present at about 200 kDa in the cell lysates (Figure 2D). Next, we performed Western blot analysis on the same lysates using the serum from our patient and a control subject. We detected immunostaining of the same size proteins in both TRPM1-Flag and mTRPM1 with the patient’s serum (Figure 2D; Patient serum). The blots with the control serum did not have a significant band. These results showed that the autoantibody was reactive to both human and mouse TRPM1.

### Acute cell death in INL caused by injection of patient serum

**Figure 3:**
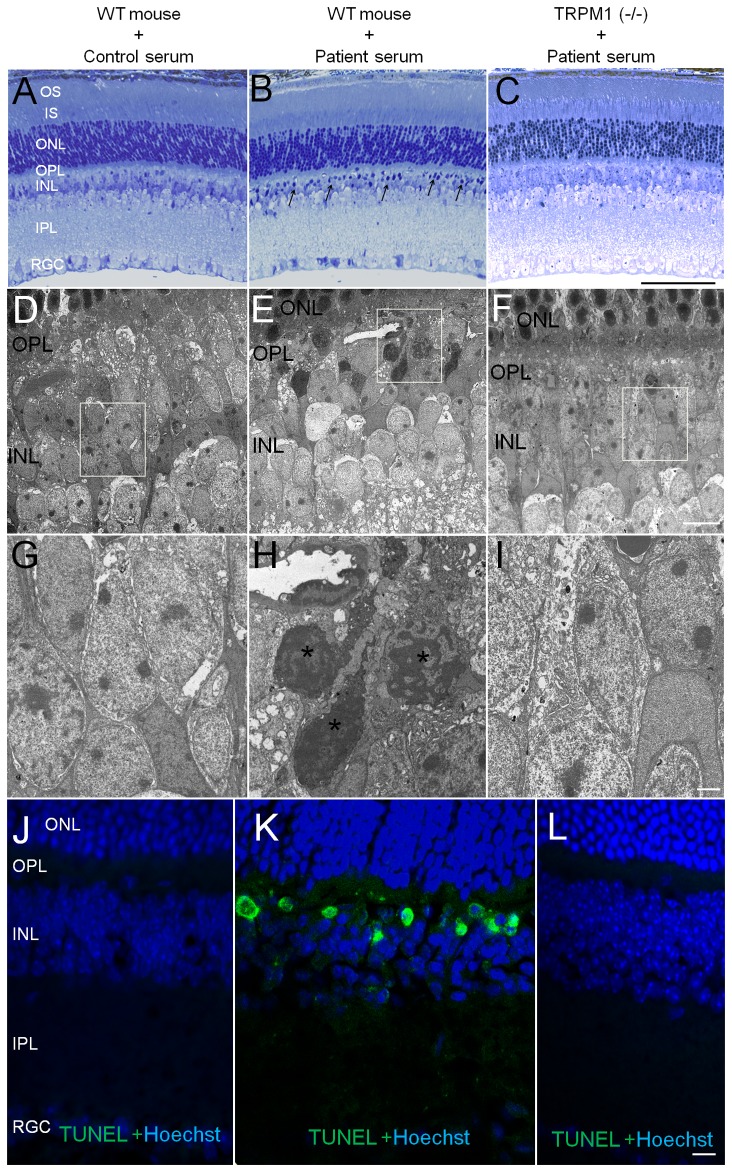
Light microscope (LM) and transmission electron microscope (TEM) photomicrographs of retinal sections. (A- C) LM photomicrographs of toluidine blue stained section obtained 5 hours after an intravitreal serum injection. (D - I) TEM photomicrographs of retinas at the same time point. (J - L) TUNEL (green) with Hoechst staining of retina obtained 1 day after the serum injection. Photomicrographs of retina from wild mouse that had the control serum injection (A, D, G, and J), the PR patient’s serum injection (B, E, H, and K) and retina from TRPM1 knockout mouse that had the patient’s serum injection. (C, F, I, and L). Photomicrographs of the regions outlined by the white boxes (D, E, and F) are enlarged below in (G, H, and I) respectively. Wild mouse retina after the injection of the patient’s serum shows many densely stained nuclei in the INL (B, arrow), and high magnification of TEM shows nuclear fragmentation and chromatin condensation of these cells (H, asterisk). Many TUNEL positive cells can be seen in the INL (K). The scale bar in the left row applies to the other two rows. The scale bar; C = 100 μm, F = 10 μm, I= 2 μm, and L = 10 μm.

Photomicrographs of the retinas of mice injected with the control serum, the patient’s serum, and a retina from a TRPM1 knockout mouse injected with the patient’s serum are shown in Figure 3. Retinal sections of eyes obtained 5 hours after the injection of the patient’s serum were examined by light microscopy (LM; Figure 3A-3C) and transmission electron microscopy (TEM; Figure 3D-3I). The toluidine blue stained retina of the mouse injected with the patient’s serum had many densely stained nuclei in the inner nuclear layer (INL) especially on the photoreceptor side (Figure 3B, arrows). We also examined the INL for any abnormalities by TEM. Low magnification TEM showed densely stained nuclei located on the photoreceptor side of the INL (Figure 3B). High magnification TEM showed nuclear fragmentation and chromatin condensation in these nuclei (Figure 3H; asterisk). LM and TEM showed no obvious abnormalities in the INL of the retina of the two other types of retinas (Figure 3A, 3C, 3D, 3F, 3G, and 3I). 

TUNEL staining was performed on retinas obtained 1 day after the serum injection (Figure 3J-3L). TUNEL-positive cells were detected in the INL where both toluidine blue stain and TEM showed abnormally stained nuclei (Figure 3 K). The number of TUNEL-positive cells/INL cells was 15.5 ± 8.0/162.7 ± 16.5 (mean ± SD) in the retina injected with the patient’s serum. In contrast, the retina of two other groups did not have any TUNEL-positive cells in the INL (Figure 3J and 3L). Thus, the absence of TUNEL-positive cells in the TRPM1 knockout mice supported the conclusion that the autoantibody against TRPM1 was implicated in cell death. 

### Loss of bipolar histochemical and structural markers after injection of patient serum

**Figure 4:**
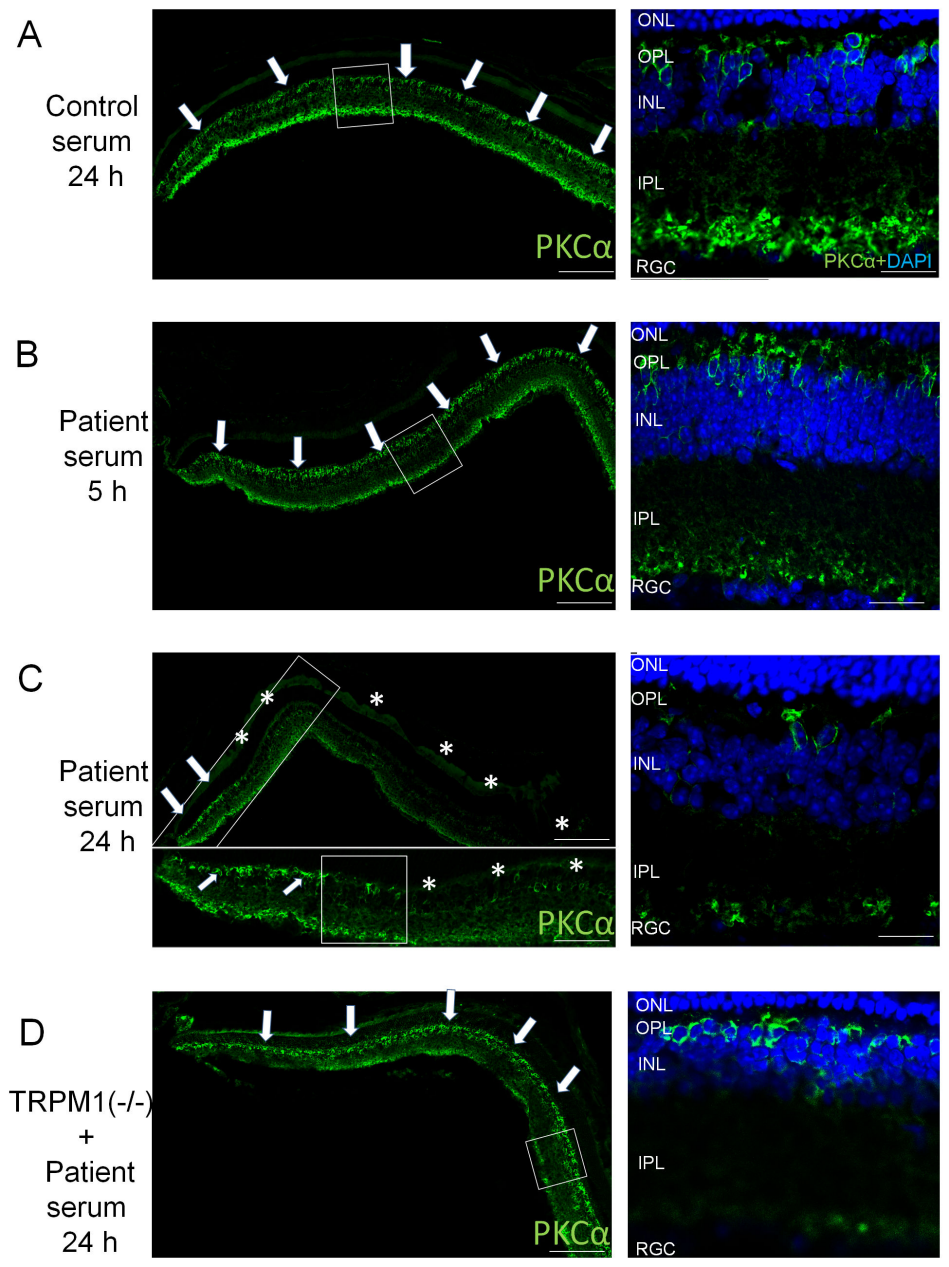
Distribution of ON bipolar cell marker (PKCα) in the retina after serum injection. Wild type mouse retina 24 hours after control serum injection (A) and wild type mouse retina 5 hours (B) and 24 hours (C) after intravitreal injection with the patient’s serum. Retina from TRPM1 knockout mouse 24 hours after intravitreal injection with the patient’s serum.(D) Mouse retinas were stained with anti PKCα antibody (green) and co-stained with DAPI (blue) in the high magnification micrographs (A-C, right). Photomicrographs of the regions outlined by the white boxes are enlarged either to the right or the below the original images. The peripheral retina is oriented to the left and the central retina to the right. PKCα staining can be seen in the entire retina at both 5 hours after the patient serum injection and 24 hours after control serum injection (A and B, arrows). But the PKCα staining is mainly absent 24 hours after the injection of the patient’s serum (C, asterisk) and remained in only the peripheral retina (C, arrows). PKCα staining can be seen 24 hours after the injection of the patient’s serum in TRPM1 knockout mouse retina (D arrows). High magnification micrograph showed that the PKC α positive cell bodies were located mainly on the photoreceptor side of the INL. The scale bars are: 60 µm for A left, B left, C upper left and D left; 20 µm for A, B, C and D right; 30 μm for C lower left.

To determine the distribution of ON bipolar cells, we performed immunohistochemical analysis using antibody against protein kinase C alpha subunit (PKCα) which is a marker of rod ON bipolar cells [26]. PKCα is located in the cell bodies, the dendrites, and the synaptic terminals of rod ON bipolar cells [27,28]. High magnification photomicrographs showed that the PKC α-positive cell bodies were located mainly on the photoreceptor side of the INL where the condensation of nuclei was found (Figure 3B). PKCα staining was present in the retina at 5 hours after the patient’s serum injection and at 24 hours after control serum injection (Figure 4A and 4B, arrows). However, PKCα staining was absent in most of the retina excluding the periphery at 24 hours after the injection of patient serum, (Figure 4C, arrows). These results supported the conclusion that PKCα was down-regulated or the ON bipolar cells had degenerated. Conversely, PKCα staining was found in TRPM1 KO mouse even at 24 hours after the injection of the patient’s serum (Figure 4D, asterisk). These results indicated that PKCα staining was absent because of the interaction between the patient’s sera and TRPM1 protein. We evaluated 3 retinas from each time point and obtained the same results. 

**Figure 5:**
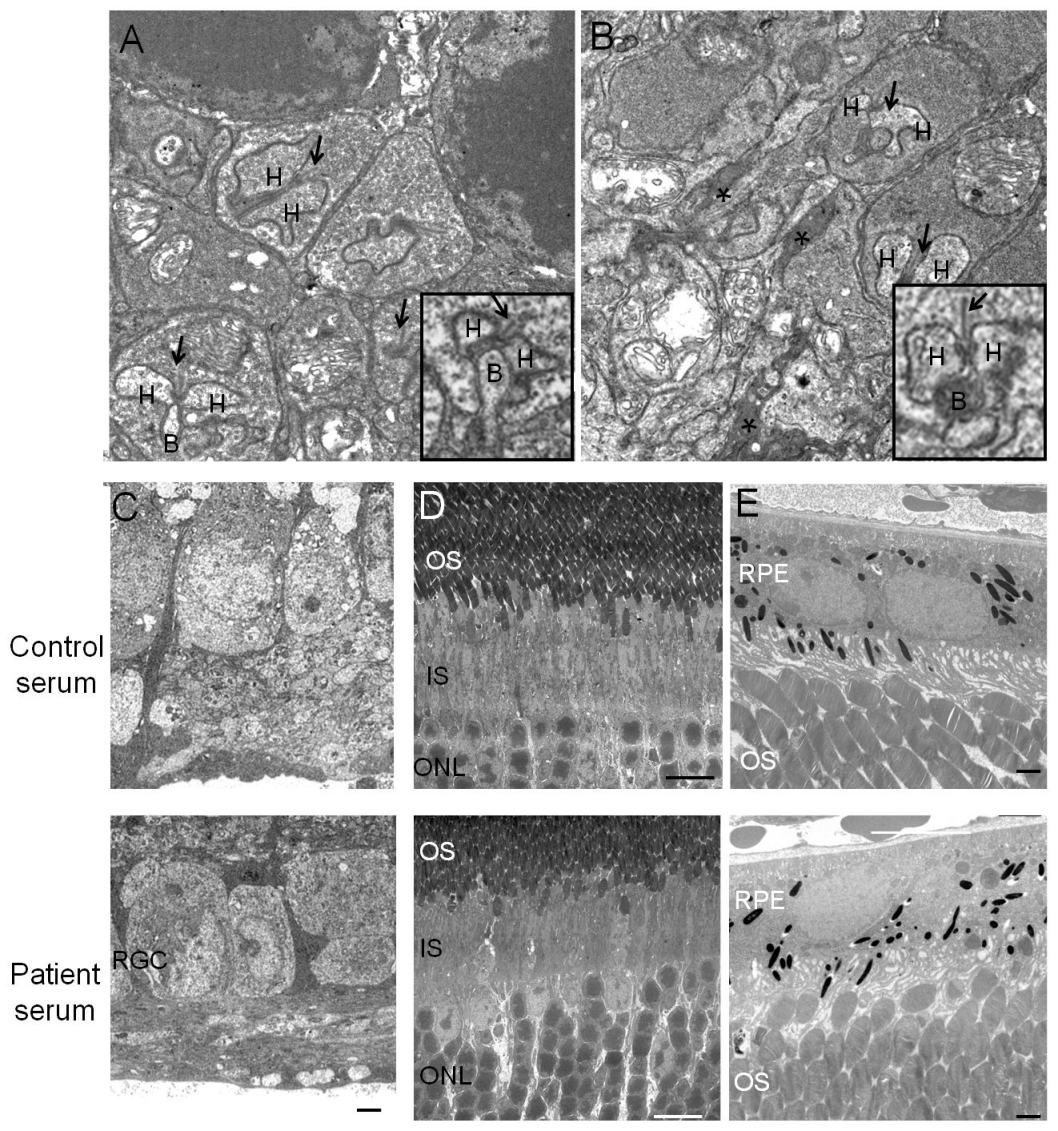
Ultrastructure of other parts of the retina 5 hours after serum injection. (A ,B) Photomicrographs of synaptic terminals between photoreceptors and rod ON bipolar cells are shown. Wild mouse retina injected intravitreally with control serum (A) or with patient's serum (B). The arrows point to the photoreceptor synaptic ribbons. The photoreceptor synaptic ribbons are surrounded by dendrites of two horizontal cells and one invaginating rod ON bipolar cell (4A,insertion). After the injection of the patient’s serum, invaginated rod ON bipolar cell dendritic terminals that extended to ribbon synapses were darkly stained (B, asterisk and insertion). (C, D, and E) After the patient’s serum injection (lower), the retinal ganglion cells (RGCs), ONL, inner segments of photoreceptors(IS), outer segments of photoreceptors (OS), and retinal pigment epithelium (RPE) showed no abnormalities compared with the retina treated with control serum (upper). The scale bar; A and B = 500 nm; C and E = 2 μm; D = 10 μm. Abbreviations: H - horizontal cell, B - ON bipolar cells.

We then examined the ultrastructure of the OPL and especially the synapses between the photoreceptors and ON bipolar cells. Dendrites of the retinal ON bipolar cells reached the ribbon synapses of photoreceptors. We focused on the abnormalities of the dendrites of retinal rod ON bipolar cells. Photomicrographs of the OPL of a mouse 5 hours after the injection of the control serum or patient’s serum are shown in Figures 5A and 5B, respectively. In the retina of the mouse that received the control serum, the photoreceptor synaptic ribbons were surrounded by the dendrites of two horizontal cells and that of one invaginated rod ON bipolar cell (Figure 5A insertion) [29]. In the retina that received the patient’s serum, the invaginated rod ON bipolar cell dendritic terminal that extend to the ribbon synapse was darkly stained (Figure 5B, arrowhead). The structures in the retinal rod ON bipolar cells were darkly stained but horizontal cell were not affected (Figure 5B insertion). These observations suggested a degeneration of dendrites [30,31]. Other parts of the retina, the retinal pigment epithelium (RPE), photoreceptor outer and inner segments, ONL, and RGCs, appeared not to be affected by the patient’s serum (Figure 5C-5E). 

### Macrophages in INL three days after patient serum injection

**Figure 6:**
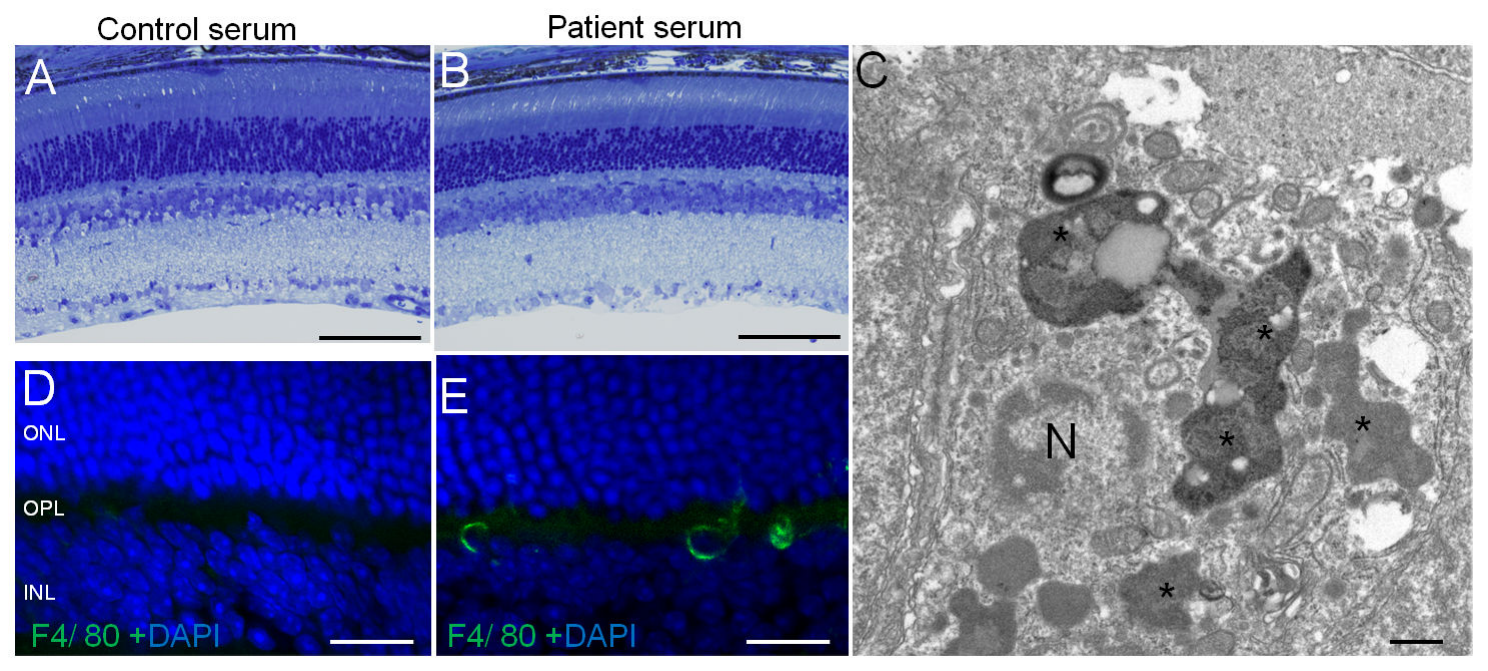
Macrophages are present in the INL 3 days after PR patient’s serum was injected. Toluidine blue staining (A and B) and TEM micrographs (C) are of a retina 3 days after the intravitreous injection of the sera. F4/80 immunostaining (green) was co-stained with DAPI (D and E). Wild mouse retina after injection of control serum (A and D) and patient's serum (B, C, and E) are shown. (B) No obvious abnormality can be seen after the patient’s serum injection. (C) TEM shows nucleus of macrophage and engulfing the apoptotic cells (N indicates the nucleus of a macrophage, asterisk indicates debris of engulfed apoptotic cells). F4/80 (green) positive cell can be seen in the INL (E). The scale bars; (A) and (B) = 100 μm; (C) = 500 nm; (D) and (E) = 20 μm.

Toluidine blue stained retinal sections obtained 3 days after the injection of control serum and patient’s serum are shown in Figures 6A and 6B, respectively. Nuclear condensation was observed 5 hours after the patient serum injection (Figure 3 B) but none was present 3 days after the injection (Figure 6B). When the INL was examined by TEM, macrophages were found surrounding the apoptotic cells (Figure 6C). F4/80-positive macrophages were detected (Figure 6E) in the layer in which TUNEL-positive cells were detected in Figure 3K. But the number of F4/80-positive cells was low, and we were able to detect only 1 to 2 cells/field (180 x 240 μm) in the immunohistochemical examinations. These results suggested that macrophages and/or other phagocytic cells had probably cleared the apoptotic cells detected in Figure 3B.

### Progressive loss of bipolar cells detected 3 months after patient serum injection

**Figure 7:**
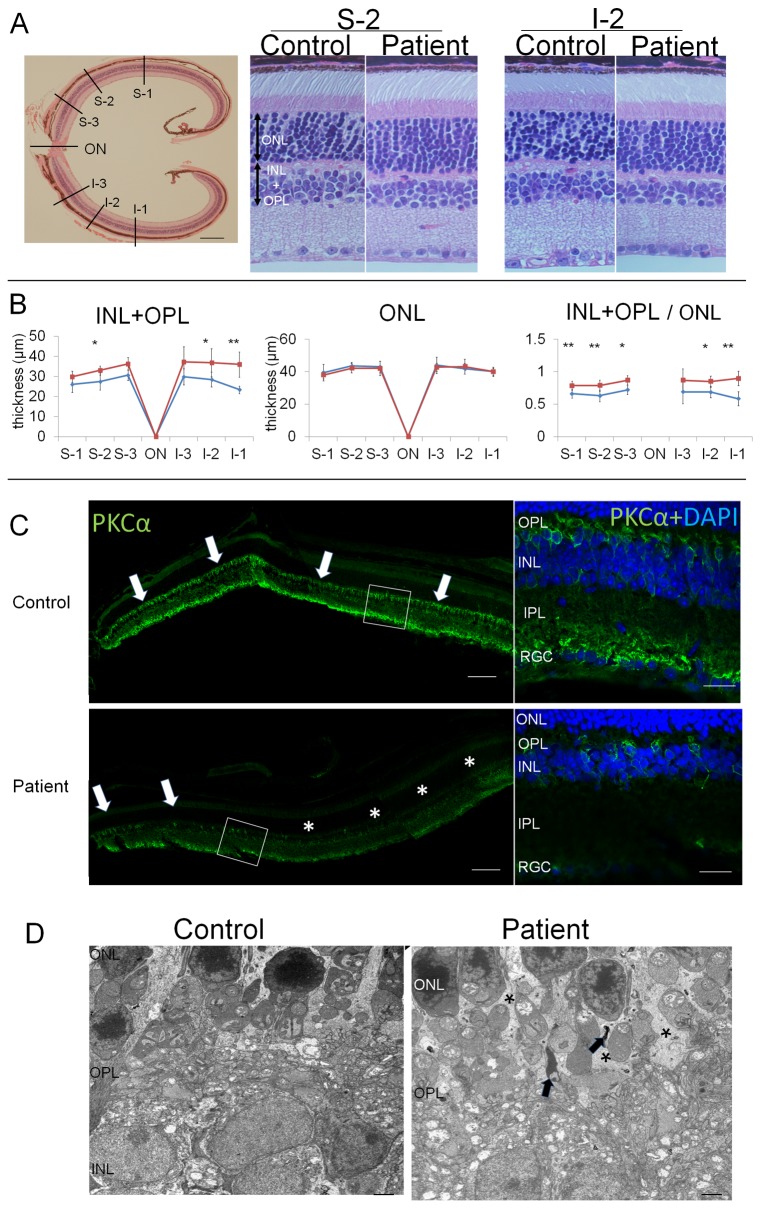
Retina obtained 3 months after the serum injection. (A) HE staining of retina from a mouse that received an intravitreal injection of control or patient's serum 3 months earlier. The thickness of the different layers was measured every 400 μm across both the superior (S1-3) and inferior hemispheres (I1-3) as shown in the low magnification micrograph on the left. Two locations of the retinas are shown (right side of A). The measurement points of the INL+OPL and the ONL are shown by the arrows. The thickness of the INL+OPL, the ONL, and the ratio of INL+OPL/ ONL are shown in (B) (n = 6). The data are the means ± SDs. (*P <0.05 and **P <0.01). . Distribution of ON bipolar cell marker (PKCα) in the retina 3 months after the injection of serum from a control subject or from the PR patient (C). Photomicrographs of the regions outlined by the white boxes are enlarged to the right. PKCα staining can be seen in the entire retina after the serum from the control subject (C upper, arrows). But PKC staining was mostly absent 3 months after the injection of the patient’s serum (C lower, asterisk) and remained only in the peripheral retina (C lower, arrows). (D) Ultrastructure of OPL 3 months after serum injection. The retina of the mouse that received an intravitreal injection of the control serum (left) and the patient's serum (right) are shown. Some of neuropils in the OPL were absent (D right, asterisk) and some debris (D left, arrows) can be seen in the retina after the injection of the patient’s serum. The scale bar; A, left = 150 μm; A, right = 20 μm; C, left = 60 μm; C, right = 15 μm; D = 2 μm.

The HE stained retinas obtained 3 months after the patient’s serum had neither nuclear condensation which was observed 5 hours after injection in INL nor distortion of retinal layers including ONL, INL and RGC compared to the retina after control serum injection (Figure 7A). We suggest that this was because of the phagocytosis of the apoptotic cells in the INL (Figure 6). To confirm this, we measured the thickness of INL and the OPL of the two groups of mice; the INL is where the ON bipolar cell bodies are located and the OPL where the dendrites are located. Because the OPL was too thin to measure, we evaluated the INL+OPL. We also measured the thickness of the ONL as shown in Figure 7A. The location 400μm , 800μm and 1200 superior to the optic disc were defined as S-3,S-2, and S-1 respectively, and the location 400μm , 800μm and 1200 inferior to the optic disc were defined I-3,I-2, and I-1 respectively. The combined INL+OPL in the mice injected with the patient’s serum was significantly thinner than that in mice injected control serum at S-2, I-2, and I-1. However, the thickness of the ONL was not significantly different between the two groups at any measured points. To avoid the effects of cutting biases, i.e., not sectioning in the vertical plane, we also checked the ratio of INL+OPL/ONL. The ratios of INL+OPL/ONL were significantly different between the two groups at all sites except I-3. These results supported the conclusion that ON bipolar cells were lost. 

We also examined the distribution of ON bipolar cells 3 months after the injection of the serum (Figure 7C). PKCα-positive staining was found over the entire retina 3 months after the control serum injection (Figure 7C upper, arrows). On the other hand, PKCα-positive staining was observed only in the peripheral retina (Figure 7C lower, arrows). These results are similar to those at 24 h after the injection and indicated that the ON bipolar cells degenerated soon after the patient serum injection, and they did not regenerate.

We also examined the OPL by TEM (Figure 7D). The neuropil of the OPL was less dense compared to that of the retina that received control serum (Figure 7D right, asterisk). Some cellular debris was found in the OPL of the patient’s serum injected retina (Figure 7D right, arrows). 

## Discussion

### Comparisons with clinical findings in paraneoplastic retinopathy

Our results showed that the intravitreal injection of serum containing an autoantibody against TRPM1 caused ON bipolar cell degeneration within 5 hours. Because the antigen for the autoantibody against ON bipolar cells has not been identified until recently, the mechanism of the ON bipolar cell dysfunction in patients with PR was not known. Two LM histopathological studies of postmortem retinas of MAR patients had conflicting results; one reported no anatomic abnormalities throughout the retina [6] and the other reported a marked reduction in the number of nuclei in the INL [32]. Even if the ON bipolar cells of PR patients degenerate as they did in our mouse model, we believe that it would be difficult to detect the changes by light microscopy because the cellular organization of retina obtained 3 months after the serum injections appeared almost normal by light microscopy (Figure 7A). However, we did find that the thickness of the combined INL+OPL of the mouse retina treated with the patient’s serum was thinner than that of control (Figure 7B). Because the difference was slight, it would have been difficult to draw a conclusion from one MAR patient because the thickness of human retina is variable. 

Lei and colleagues reported that an intravitreal injection of purified IgG from a MAR patient into monkey eyes led to a reduction in the amplitude of the photopic ERG b-waves [33]. They also reported that the reduction of the b-wave was transient, and the b-wave recovered 3 months after the IgG injection. They also reported injection of the MAR serum into rodent eyes (rat and guinea pig) had no effect on the ERG. We cannot explain the difference from our findings but we suggest that the mechanism for the ON bipolar cell dysfunction is probably different from that of our mice because our results showed a permanent damage. This discrepancy may also be because of the difference in the species examined, concentration of the antibody, relative size of the eye, and injected volume. Another more likely possibility was that the antigen of the IgG was not TRPM1. An antibody against TRPM1 was found in only about 10% of MAR patients in one study [11], and in 2 of 3 MAR patients in another study [10]. These reports indicated that there may be other antigens that can cause MAR in some patients. 

There is a report that the signs and symptoms improve in some MAR patients after therapy [6] and other antigens of IgG may have induced the transient ON bipolar dysfunction in these patients. We suggest that an autoantibody against TRPM1 may not apply to all the cases with this syndrome [6]. 

### ERGs of patient and serum-injected mice

The ERGs recorded after APB-injected mice resembled those of mice after the injection of the PR patient’s serum; under scotopic and photopic condition, the ERG b-waves were markedly reduced and the amplitude of scotopic a-wave was almost normal. Because it is known that APB blocks ON bipolar cell activity, these results suggested a dysfunction of the ON bipolar cells after the patient’s serum injection. 

Another retinal disease with ON bipolar cell dysfunction is the complete type of congenital stationary night blindness (cCSNB). The ERGs recorded from patients with cCSNB are similar to those recorded from patients with PR, viz., a marked reduction of the b-wave under scotopic conditions and preserved b-wave under photopic conditions. cCSNB is caused by mutations in the genes that mediate the transduction cascade of the ON bipolar cells, and the symptoms and retinal changes do not change throughout life [34-37]. Mouse models for cCSNB do not have obvious changes in the retinal cellular organization but there is a functional loss of ON bipolar cells. The waveforms of the mouse ERGs in the eyes that received the patient’s serum resembled those of the mouse model of cCSNB [25,38]. Under photopic conditions, there is a difference in the shape of the ERGs between human and mice that have ON bipolar cell dysfunction. This difference was most likely present because the mouse photopic b-waves originate mainly from ON bipolar cells but the human photopic b-waves originate from both the ON and OFF bipolar cells [39]. Thus, the positive OFF response remains even after the loss of the ON bipolar cell response. The shared features of the ERGs between the patients and the mice injected with the patient’s serum suggest that the two species also share the pathomechanism of the ON bipolar cell dysfunction. But we have to consider the differences in the immune responses between humans and mice because the mouse retina injected with human IgG and the patient retina affected by human IgG may respond differently. 

### Apoptotic cells in inner nuclear layer

The most interesting finding of this study was that the serum-containing autoantibody killed the ON bipolar cells very quickly. Our results showed that some of the inner nuclear cells were degenerated as early as 5 hours after the intravitreal injection of the patient’s serum (Figure 3), and apoptotic cell death was detected by TUNEL staining at 1 day postinjection. Electon microscopy (Figure 3E and H) showed nuclear condensation which indicated apoptosis of these cells. Because there are many different types of cells in the INL, e.g., ON bipolar cells, OFF bipolar cells, Mueller cells, horizontal cells, and amacrine cells, it was difficult to determine which type was the apoptotic cell. We were not able to show that the TUNEL positive cells were the retinal ON bipolar cells by double labeling with ON bipolar cell maker (PKCα) because the ON bipolar cells had already disappeared (see Figure 4) by 1 day postinjection. In addition, 5 hours was too early to detect TUNEL positive cells. However, we found that the apoptotic cells were located on the photoreceptor side of the INL which is where the ON bipolar cell nuclei are located (Figure 4) [27,40]. In addition, the dendritic tips that invaginated into the photoreceptor synapses were the ones that were darkly stained. Because this is the location of the ON bipolar cell dendritic tips, our findings indicated that the ON bipolar cells were degenerated (Figure 4). Thus, we conclude that the apoptotic cells in the INL were ON bipolar cells, and the negative waveform of the ERGs and extinguished ON bipolar cell marker supported this. 

### Removal of degenerated ON bipolar cells

Macrophages were found surrounding the apoptotic cells in the INL (Figure 5C). In a mouse model of retinal detachment, the degenerated photoreceptors were removed by macrophages [41]. In our model, the degenerated bipolar cells were probably also removed in the same way. After the clearance of the apoptotic bipolar cells, the retinal architecture appeared to be well organized except for a thinning of the INL+OPL. Because the ERGs did not recover even at 6 months after the injection of the serum and most of the rod ON bipolar cell maker (PKCα) was absent at 3 months after injection, the rod ON bipolar cells most likely did not regenerate. Thus, we conclude that the reduction of the b-wave was due to the loss of ON-bipolar cell, and the change is permanent. 

### Comparisons of eye manifestations in patients and mice

Patients with PR present with acute night blindness, and our results in mice suggest that the acute apoptosis of the ON bipolar cells may have been the cause of the symptom and signs of PR patients. Optical coherence tomography (OCT) showed that the morphology of the patient’s retina was normal [11]. These OCT data indicate that the alterations in the morphology of the patient’s retina might be very slight as we showed in the light microscopic images of the retina with almost normal appearances 3 months after the patient serum injection (Figure 7A). 

Our PR patient had chemotherapy and now is in complete remission but his symptoms and ERG changes have not recovered. Our morphological data suggest degenerated ON bipolar cells did not regenerate. In patients, fluorescein angiography showed that PR patients have leakage from the retinal blood vessels [11], and it is likely that the autoantibodies leaked out to reach the ON bipolar cells. Thus, we suggest that antibodies directly affected the TRPM1 channels of the ON bipolar cell in both the patient and mice. 

The reason we were able to detect ON bipolar cell dysfunction in mice similar to that in PR patients may be because TRPM1 is a membrane protein and the autoantibody easily recognizes the antigen. We believe that our animal model system will be useful for analyzing the mechanism of PR, especially in the cases antigens that are membrane proteins. 

Our study has some limitations. We used the serum of just one PR patient, however only six PR patients who have the TRPM1 autoantibody have been reported worldwide. Thus, it would be interesting to investigate the effect of other autoantibodies found in these PR patients.

## Conclusions

Our results show that an intravitreal injection of the serum of a patient with PR into the vitreous of mice led to alterations of the ERG that resembled those of the patient with PR. Histological analysis showed that the ON bipolar cells die by apoptosis. As best as we can determine, there has been no previous report of a specific degeneration of retinal ON bipolar cells in hereditary or acquired retinal diseases. 

## References

[1] Sawyer RA, Selhorst JB, Zimmerman LE, Hoyt WF (1976) Blindness caused by photoreceptor degeneration as a remote effect of cancer. Am J Ophthalmol 81: 606-613. PubMed: 179323. 179323 10.1016/0002-9394(76)90125-2

[2] Thirkill CE, Tait RC, Tyler NK, Roth AM, Keltner JL (1992) The cancer-associated retinopathy antigen is a recoverin-like protein. Invest Ophthalmol Vis Sci 33: 2768-2772. PubMed: 1388144. 1388144

[3] Milam AH, Saari JC, Jacobson SG, Lubinski WP, Feun LG et al. (1993) Autoantibodies against retinal bipolar cells in cutaneous melanoma-associated retinopathy. Invest Ophthalmol Vis Sci 34: 91-100. PubMed: 8425845. 8425845

[4] Heckenlively JR, Ferreyra HA (2008) Autoimmune retinopathy: A review and summary. Semin Immunopathol 30: 127-134. doi:10.1007/s00281-008-0114-7. PubMed: 18408929. 18408929

[5] Jacobson DM, Adamus G (2001) Retinal anti-bipolar cell antibodies in a patient with paraneoplastic retinopathy and colon carcinoma. Am J Ophthalmol 131: 806-808. doi:10.1016/S0002-9394(00)00925-9. PubMed: 11384586. 11384586

[6] Keltner JL, Thirkill CE, Yip PT (2001) Clinical and immunologic characteristics of melanoma-associated retinopathy syndrome: Eleven new cases and a review of 51 previously published cases. J Neuroophthalmol 21: 173-187. doi:10.1097/00041327-200109000-00004. PubMed: 11725182. 11725182

[7] Berson EL, Lessell S (1988) Para-neoplastic night blindness with malignant-melanoma. Am J Ophthalmol 106: 307-311. doi:10.1016/0002-9394(88)90366-2. PubMed: 2971322. 2971322

[8] Alexander KR, Fishman GA, Peachey NS, Marchese AL, Tso MOM (1992) On response defect in paraneoplastic night blindness with cutaneous malignant-melanoma. Invest Ophthalmol Vis Sci 33: 477-483. PubMed: 1544774. 1544774

[9] Goetgebuer G, Kestelyn-Stevens A-M, De Laey J-J, Kestelyn P, Leroy BP (2008) Cancer-associated retinopathy (CAR) with electronegative ERG: a case report. Doc Ophthalmol 116: 49-55. doi:10.1007/s10633-007-9074-9. PubMed: 17721792. 17721792

[10] Dhingra A, Fina ME, Neinstein A, Ramsey DJ, Xu Y et al. (2011) Autoantibodies in melanoma-associated retinopathy target TRPM1 cation channels of retinal ON bipolar cells. J Neurosci 31: 3962-3967. doi:10.1523/JNEUROSCI.6007-10.2011. PubMed: 21411639. 21411639 PMC3073846

[11] Kondo M, Sanuki R, Ueno S, Nishizawa Y, Hashimoto N et al. (2011) Identification of autoantibodies against TRPM1 in patients with paraneoplastic retinopathy associated with ON bipolar cell dysfunction. PLoS One e19116: e19911 21611200 10.1371/journal.pone.0019911 PMC3096646

[12] Zimov S, Yazulla S (2004) Localization of vanilloid receptor 1 (TRPV1/VR1)-like immunoreactivity in goldfish and zebrafish retinas: restriction to photoreceptor synaptic ribbons. J Neurocytol 33: 441-452. doi:10.1023/B:NEUR.0000046574.72380.e8. PubMed: 15520529. 15520529

[13] Morgans CW, Zhang J, Jeffrey BG, Nelson SM, Burke NS et al. (2009) TRPM1 is required for the depolarizing light response in retinal ON-bipolar cells. Proc Natl Acad Sci U S A 106: 19174-19178. doi:10.1073/pnas.0908711106. PubMed: 19861548. 19861548 PMC2776419

[14] Koike C, Obara T, Uriu Y, Numata T, Sanuki R et al. (2010) TRPM1 is a component of the retinal ON bipolar cell transduction channel in the mGluR6 cascade. Proc Natl Acad Sci U S A 107: 332-337. doi:10.1073/pnas.0912730107. PubMed: 19966281. 19966281 PMC2806705

[15] Rogers SW, Andrews PI, Gahring LC, Whisenand T, Cauley K et al. (1994) Autoantibodies to glutamate-receptor glur3 in rasmussens encephalitis. Science 265: 648-651. doi:10.1126/science.8036512. PubMed: 8036512. 8036512

[16] Whitney KD, McNamara JO (2000) GluR3 autoantibodies destroy neural cells in a complement-dependent manner modulated by complement regulatory proteins. J Neurosci 20: 7307-7316. PubMed: 11007888. 11007888 10.1523/JNEUROSCI.20-19-07307.2000 PMC6772766

[17] Dalmau J, Gleichman AJ, Hughes EG, Rossi JE, Peng X et al. (2008) Dessain SK, Rosenfeld MR, Balice-Gordon R, Lynch DR: Anti-NMDA-receptor encephalitis: case series and analysis of the effects of antibodies. Lancet Neurol 7:1091-1098 10.1016/S1474-4422(08)70224-2 PMC2607118 18851928

[18] Slaughter MM, Miller RF (1981) 2-amino-4-phosphonobutyric acid: a new pharmacological tool for retina research. Science 211: 182-185. 6255566 10.1126/science.6255566

[19] Ueno S, Kondo M, Miyata K, Hirai T, Miyata T et al. (2005) Physiological function of S-cone system is not enhanced in rd7 mice. Exp Eye Res 81: 751-758. doi:10.1016/j.exer.2005.04.013. PubMed: 16005871. 16005871

[20] Bush RA, Sieving PA (1994) A proximal retinal component in the primate photopic ERG a-wave. Invest Ophthalmol Vis Sci 35: 635-645. PubMed: 8113014. 8113014

[21] Ueno S, Kondo M, Niwa Y, Terasaki H, Miyake Y (2004) Luminance dependence of neural components that underlies the primate photopic electroretinogram. Invest Ophthalmol Vis Sci 45: 1033-1040. doi:10.1167/iovs.03-0657. PubMed: 14985327. 14985327

[22] Brown KT (1969) The electroretinogram: its components and their origins. UCLA Forum Med Sci 8: 319-378. PubMed: 4990860. 4990860

[23] Granit R (1933) The components of the retinal action potential in mammals and their relation to the discharge in the optic nerve Part I Isolation of components in the retinal action potential of the dark-adapted decerebrate preparation. J Physiol-London 77: 207-239. PubMed: 16994385. 16994385 10.1113/jphysiol.1933.sp002964 PMC1394767

[24] Machida S, Kondo M, Jamison JA, Khan NW, Kononen LT et al. (2000) P23H rhodopsin transgenic rat: Correlation of retinal function with histopathology. Invest Ophthalmol Vis Sci 41: 3200-3209. PubMed: 10967084. 10967084

[25] Masu M, Iwakabe H, Tagawa Y, Miyoshi T, Yamashita M et al. (1995) Specific deficit of the on response in visual transmission by targeted disruption of the mGluR6 gene. Cell 80: 757-765. doi:10.1016/0092-8674(95)90354-2. PubMed: 7889569. 7889569

[26] Grünert U, Martin PR, Wässle H (1994) Immunocytochemical analysis of bipolar cells in the macaque monkey retina. J Comp Neurol 348: 607-627. doi:10.1002/cne.903480410. PubMed: 7530731. 7530731

[27] Ruether K, Feigenspan A, Pirngruber J, Leitges M, Baehr W et al. (2010) PKC alpha Is Essential for the Proper Activation and Termination of Rod Bipolar Cell. Response - Invest Ophthalmol Vis Sci 51: 6051-6058. doi:10.1167/iovs.09-4704. 20554612 PMC3261049

[28] Kolb H, Zhang L, Dekorver L (1993) Differential staining of neurons in the human retina with antibodies to protein kinase C isozymes. Vis Neurosci 10: 341-351. doi:10.1017/S0952523800003734. PubMed: 8485096. 8485096

[29] Vardi N (1998) Alpha subunit of G(0) localizes in the dendritic tips of ON bipolar cells. J Comp Neurol 395: 43-52. doi:10.1002/(SICI)1096-9861(19980525)395:1. PubMed: 9590545. 9590545

[30] Ichikawa M, Arissian K, Asanuma H (1985) Distribution of corticocortical and thalamocortical synapses on identified motor cortical neurons in the cat: Golgi, electron microscopic and degeneration study. Brain Res 345: 87-101. doi:10.1016/0006-8993(85)90839-X. PubMed: 2998551. 2998551

[31] Leong SK, Wong WC (1989) An ultrastructural study of the stellate ganglion of the pig-tailed monkey (Macaca nemestrina). J Anat 164: 1-18. PubMed: 2606786. 2606786 PMC1256594

[32] Gittinger JW Jr., Smith TW (1999) Cutaneous melanoma-associated paraneoplastic retinopathy: histopathologic observations. Am J Ophthalmol 127: 612-614. doi:10.1016/S0002-9394(98)00431-0. PubMed: 10334362. 10334362

[33] Lei B, Bush RA, Milam AH, Sieving PA (2000) Human melanoma-associated retinopathy (MAR) antibodies alter the retinal ON-response of the monkey ERG in vivo. Invest Ophthalmol Vis Sci 41: 262-266. PubMed: 10634629. 10634629

[34] Miyake Y, Yagasaki K, Horiguchi M, Kawase Y, Kanda T (1986) Congenital stationary night blindness with negative electroretinogram - A new classification. Arch Ophthalmol 104: 1013-1020. doi:10.1001/archopht.1986.01050190071042. PubMed: 3488053. 3488053

[35] Bech-Hansen NT, Naylor MJ, Maybaum TA, Sparkes RL, Koop B et al. (2000) Mutations in NYX, encoding the leucine-rich proteoglycan nyctalopin, cause X-linked complete congenital stationary night blindness. Nat Genet 26: 319-323. doi:10.1038/81619. PubMed: 11062471. 11062471

[36] Dryja TP, McGee TL, Berson EL, Fishman GA, Sandberg MA et al. (2005) Night blindness and abnormal cone electroretinogram ON responses in patients with mutations in the GRM6 gene encoding mGluR6. Proc Natl Acad Sci U S A 102: 4884-4889. doi:10.1073/pnas.0501233102. PubMed: 15781871. 15781871 PMC555731

[37] Pusch CM, Zeitz C, Brandau O, Pesch K, Achatz H et al. (2000) The complete form of X-linked congenital stationary night blindness is caused by mutations in a gene encoding a leucine-rich repeat protein. Nat Genet 26: 324-327. doi:10.1038/81627. PubMed: 11062472. 11062472

[38] Pardue MT, McCall MA, LaVail MM, Gregg RG, Peachey NS (1998) A naturally occurring mouse model of X-linked congenital stationary night blindness. Invest Ophthalmol Vis Sci 39: 2443-2449. PubMed: 9804152. 9804152

[39] Sieving PA, Murayama K, Naarendorp F (1994) Push-pull model of the primate photopic electroretinogram - A role for hyperpolarizing neurons in shaping the b-wave. Vis Neurosci 11: 519-532. doi:10.1017/S0952523800002431. PubMed: 8038126. 8038126

[40] Jeon CJ, Strettoi E, Masland RH (1998) The major cell populations of the mouse retina. J Neurosci 18: 8936-8946. PubMed: 9786999. 9786999 10.1523/JNEUROSCI.18-21-08936.1998 PMC6793518

[41] Hisatomi T, Sakamoto T, Sonoda KH, Tsutsumi C, Qiao H et al. (2003) Clearance of apoptotic photoreceptors - Elimination of apoptotic debris into the subretinal space and macrophage-mediated phagocytosis via phosphatidylserine receptor and integrin alpha v beta 3. Am J Pathology 162: 1869-1879. doi:10.1016/S0002-9440(10)64321-0. PMC1868143 12759244

